# Proxy consent to clinical research participation: how should it be justified?

**DOI:** 10.1007/s11019-026-10326-6

**Published:** 2026-02-14

**Authors:** Julius Sim, Anthony Wrigley

**Affiliations:** 1 School of Medicine, Keele University, Staffordshire, ST5 5BG UK; 2 School of Law, Keele University, Staffordshire, ST5 5BG UK

**Keywords:** Proxy consent, Surrogates, Ethics, Clinical research, WD Ross, Intuitionism

## Abstract

In situations where first-hand, contemporaneous consent cannot be obtained from potential research participants—such as from those who lack competence—consent may be sought from a proxy, such as a family member. Such proxy consent must be shown to have a sound moral justification if it is to be an acceptable alternative to first-hand consent. Two standards traditionally proposed for this purpose are those of substituted judgment and best interests. We describe and discuss the limitations of these two approaches, with particular reference to ways in which the context of research differs from that of clinical practice, where proxy consent has been more widely utilized. Other approaches that expand upon or depart from these traditional justifications are then discussed, namely the authentic life, endorsed life, and substituted interests models, as well as one that grounds proxy consent in a putative obligation to participate in research. Whilst these models obviate some of the limitations of the substituted judgment and best interests approaches, they have shortcomings of their own, and do not take full account of all of the relevant values and motivations that obtain in the context of clinical research. We propose an alternative and, in our view, more fruitful approach to justifying proxy consent to research participation—based on W D Ross’s moral intuitionism—that does not rest upon a single moral principle but can accommodate a range of both deontological and consequentialist *prima facie* values.

## Introduction

Proxy consent—where surrogates provide consent on behalf of persons who are not competent to do so for themselves—is a topic that has been analysed within applied ethics by a number of authors (e.g., O’Neil 1983; Buchanan and Brock 1989; Wrigley 2007, 2015, 2018). Within medical ethics specifically, situations such as childhood disease, critical care, life-sustaining treatment, Alzheimer’s disease and other forms of mental or cognitive impairment are often the focus. In relation to research ethics, however, discussion of proxy consent has centred predominantly on empirical questions. These have addressed issues such as attitudes and beliefs regarding proxy decision-making (e.g., Ayalon 2009; De Vries et al. 2010; Dubois et al. 2011; Shepherd et al. 2019; Mahafzah et al. 2021), the dynamics of proxy consent discussions (e.g., Sugarman et al. 2007), the ‘accuracy’ of relatives’ proxy decisions (e.g., Coppolino and Ackerson 2001; Ciroldi et al. 2007), proxy decision-makers motivations for either granting or declining consent (Dunn et al. 2011), and the effect of proxy consent on trial enrolment (Mason et al. 2006). Abdoler and Wendler (2012) and Shepherd et al. (2018) have conducted syntheses of such studies. Alongside the empirical questions, there has also been a parallel concern with the legal and regulatory status of proxies in research (Kim et al. 2004; Saks et al. 2008).

Normative issues concerning proxy consent to clinical research are, however, less developed, and often rely on the more general work done in applied and medical ethics to provide the ethical foundations. This we consider to be an important omission, primarily because the research setting generates some ethically relevant contexts of its own that challenge any assumption that the basis for proxy consent in one area, such as clinical medicine, can be transferred unproblematically to another area, such as research. We address this lack of specific normative consideration here by considering the ethical status of proxy consent in the research setting. In doing so, we will assume that the individual for whom proxy consent is given, or declined, is wholly incompetent and can therefore make no contribution whatsoever to the consent decision at this time.^1^ Also underlying our discussion is a more general assumption that, to the extent that consent is centred principally in the notion of autonomy (Beauchamp and Childress 2009), the stringency of the requirement for consent is greatest in situations where the implications for an individual’s autonomous decision-making and actions are greatest. Correspondingly, where little is at stake in terms of autonomy, the requirement for consent is lessened accordingly. Similarly, the consent requirement will have greatest stringency where the welfare of the individual is potentially most at risk. It follows from this that consent is not an absolute moral requirement and may be overridden by countervailing considerations—grounded in other moral principles—that may be considered more pressing in the situation concerned.

Our position, after considering various justifications for the role of proxy consent in research, is that an ethical model that embraces an array of values and motivations involving consequentialist reasoning together with deontological duties, akin to the intuitionist approach to ethics adopted by Ross (1930, 1939), should be the preferred approach. This allows the proxy to consider a range of relevant values, including both duties to the individual and duties of broader benefit to society, through a balancing of the stringency of consent requirements and proportionality of the nature of the research activity. We end by offering a short case example as to how this might work in practice.

## Proxy consent

That there are huge benefits to individuals and society that arise from research taking place is unquestionable. Such clear potential benefit in terms of advancing knowledge, understanding, treatments, etc. does not, of course, preclude that there is also potential for significant harms to arise from research. Research is often viewed as placing much of the burden of risk of harm on the shoulders of the research participants themselves. Often, but not always, this is without the potential for individual research participants to directly benefit themselves from the research. Rather, the benefits of research are often only realized at some point in the future, and may come only to future patients. An important distinction here is that between therapeutic and nontherapeutic research. In therapeutic research, the investigator intends to provide treatment to participants in the study in addition to seeking generalizable knowledge, whereas in nontherapeutic research the intention to treat the participant is lacking, with generalizable knowledge being the sole objective (Foster 2001).^2^

Given a potential asymmetry in benefit and burden of risk, the standard accepted position in research ethics is that participation requires the ‘first-hand’, contemporaneous consent of the individual, and that such consent should be voluntarily given, based on the provision of relevant information and appropriate comprehension of this information (Foster 2001; Hughes et al. 2010; O’Shea 2018).^3^ The idea behind an insistence on first-hand consent is that such consent allows research participants to judge for themselves whether they consider the burdens of participation acceptable when considered against potential benefits and any other factors, such as personal circumstances and beliefs that may also be relevant. This does not mean that all research participation requires such consent, for if demands were too high, vast areas of research might never take place. However, it is fundamental to research ethics that a clear basis is provided as to what types of research might be allowed without consent and for what reasons. Exceptions to this consent requirement are varied but tend to focus on key factors, such as when the nature and design of the research in question preclude the possibility of gaining consent (e.g., in certain studies in social psychology requiring some form of deception of the participants; Kimmel 2007), or when the burden of participation is so small that demanding individual consent appears to be disproportionate (e.g., in some observational studies of behaviours in a public place; BPS 2021). Alternatively, in some instances it may be thought that consent can appropriately be gained at another level than the individual participant—such as from a relevant community in cluster randomized trials (Sim and Dawson 2012). In individually randomized clinical trials, however, first-hand consent by individual participants is the default requirement, and this is enshrined in the many international guidelines and regulations for biomedical research, such as: the Declaration of Helsinki (WMA 2024); the CIOMS International Guidelines for Health-Related Research Involving Humans (2016); and the UNESCO Universal Declaration on Bioethics and Human Rights (2005).

This leaves research in areas where the participants have to be from groups where individual consent is not possible in a difficult position. How can important research into the effective treatments for neonatal conditions, or for illnesses rendering individuals incompetent, be carried out if these groups are automatically excluded? While some of the safeguards in place to allow research in these areas go some way to offering an ethically justified basis for participants who cannot consent to be involved in research, they are limited in scope and miss the additional safeguards, but also the freedoms of choice, that consent facilitates. Therefore, it is reasonable to ask whether we can further capture and implement some of the benefits arising from the consent process in other ways that are suitable for the research context.

One such option arises in standard treatment or other cases involving individual welfare decisions where individual consent cannot be obtained but is nonetheless thought to be necessary, and so proxy consent may be sought. Consent is obtained vicariously: decision-making is transferred to—or assumed by—some form of surrogate acting on the individual’s behalf.^4^ The circumstances may be those in which a person has not yet gained competence to consent (e.g., a neonate) or has lost such competence either temporarily or permanently (e.g., a person who is unconscious following trauma or is in a permanent vegetative state, respectively). The situation is more complicated than in the case of first-hand consent insofar as it involves a decision as to the appropriate criteria for selecting a proxy, which may also require some substantial decisions to be made in appointing a proxy and establishing what that individual’s wishes were prior to loss of competence.

Reflecting the distinction between therapeutic and nontherapeutic research, the Declaration of Helsinki states that individuals who are not competent should only be recruited if “the research is likely to either personally benefit them or if it entails only minimal risk and minimal burden” (WMA 2024:5). Similarly, the CIOMS (2016) guidelines indicate that, in respect of research on incompetent patients that has no potential individual benefits for participants, the risks involved in participation should be minimal. Here, minimal risk is defined by “comparing the probability and magnitude of anticipated harms with the probability and magnitude of harms ordinarily encountered in daily life or during the performance of routine physical or psychological examinations or tests” (CIOMS 2016:12). The Alzheimer’s Association (2015) state that research involving greater-than-minimal risk with no reasonable potential benefit to the individual should only take place if the person is capable of providing consent or had executed an advance research directive, thereby excluding proxy consent in such a situation. An earlier position statement by the American College of Physicians (1989:844) had similarly stated that “surrogates should not consent to nontherapeutic research that presents more than a minimal risk of harm or discomfort.” These statements thereby rule out proxy consent for any nontherapeutic research with greater than minimal risk of harm.^5^

In comparison to first-hand consent, proxy consent is a *faute de mieux* and, for some, it is a particularly poor substitute. Manson and O’Neill (2007:6) argue that advocates of proxy consent “come close to disregarding or short-changing the very standards to which proponents of consent requirements aspire: actual consent is set aside in favour of somebody else’s consent, or of consent that might be given under different conditions, or by somebody with different capacities.” Those who nonetheless would defend proxy consent generally regard it as authoritative in terms of one of two ‘standard’ justifications.

Under the *substituted judgment* standard of justification, the proxy attempts to replicate a decision that the individual would have made in the situation at hand, based on that person’s known or likely preferences (Brock 1993; Sulmasy 2010; Wrigley 2018). Such a strict standard of arriving at the decision is notoriously difficult to achieve, given how questionable it is as to whether a proxy’s decision can ever perfectly replicate that of the individual for whom the proxy decision is to be made (Shalowitz et al 2006; Scholten et al 2018; Wrigley 2007, 2011). There is, however, evidence that individuals are prepared to grant some leeway—i.e., a degree of departure from their stated preferences—to those who might make a proxy decision on their behalf concerning research participation (Kim et al 2009, 2013; Scholten et al 2018); therefore, expectations are likely to be low that such an exacting standard will be achieved when appointing a proxy. Alternative approaches to determining a substituted judgment are also available to proxies. For example, consent may be inferred by the proxy from previous occasions on which the incompetent person has expressed relevantly similar wishes, or from other information that suggests what those wishes might be.^6^ Where consent is inferred from such previously expressed wishes, the more recent these wishes are, the more authoritative, *ceteris paribus*, they will be, given that they are liable to potential change over time (Danis et al. 1994). In the process, an assumption is made that what a person would have *wanted* or *preferred* equates to what he or she would have *consented* to, which is a questionable assumption given that one’s choices may be underdetermined by one’s wants or preferences.^7^

It is sometimes stated (e.g., by Veatch 2003) that substituted judgments can be based on the person’s known values as well as on previous intentions and wishes. However, we will restrict our interpretation to intentions and wishes as far as possible; firstly, because values seem to be a higher-order concept, and secondly, so as not to elide substituted judgment with approaches that centre explicitly on values, such as the endorsed life approach, that we will consider in due course.

The substituted judgment approach is based on the broader principle of respect for autonomy and is essentially counterfactual, as it invokes a situation that does not obtain (i.e., one in which the person is competent to consent and does make a decision) as the basis for a judgment as to what decision the person would have made, if able to have done so. As it is well-recognized that autonomy does not require the decision made to be the wisest or most beneficial of those available (Dworkin 1988), the substituted judgment approach may (at least theoretically and without any regulatory constraint upon the type of decision that can be made) uphold autonomy at the cost of welfare considerations.

Under the *best interests* standard of justification, decisions are taken that *are* thought to support the best interests of the person, based on the broader principle of beneficence (Wierenga 1983). There is no necessary assumption under this doctrine that the decision taken is the one that, counterfactually, the person would have taken him- or herself, as such a decision might not be regarded as conducive to that person’s best interests in the way that the proxy decision-maker sees these interests. If this *were* seen as a necessary assumption, the principle of best interests would collapse into that of substituted judgment. In a similar way, the person’s best interests must not be reducible to just his or her preferences, as this would also cause best interests to collapse into substituted judgment. Moreover, in contrast to substituted judgment, there is no requirement that the individual has had, or will ever have, a level of competence sufficient for formulating autonomous desires (O’Neil 1983). For Harris (2003), a strength of the best interests doctrine is that it does not rest upon considerations of autonomous choice, and therefore does not invoke something that, *ex hypothesi*, is not directly determinable at the time of proxy decision-making.

Under these two models of justification, decisions derive their authority from meeting a certain standard, based variously upon a knowledge of the person’s previous wishes, interests, or personal characteristics. However, in the case of substituted judgment, decisions are likely to be more epistemically demanding as they are tied more closely to what the individual’s specific autonomous intentions would likely be in the situation at hand. Proxies face an ‘epistemic burden’: the information about the individual’s intentions that they need to know (or to acquire) in order to make a decision that would honour those intentions (Crutchfield and Scheall 2019). Where such intentions have not yet been formed, or cannot be known or learned, such as in the case of individuals below the age of competence, the substituted judgment approach cannot apply in the way it was intended (Freedman 1975). In contrast, the best interests approach does not necessarily relate to specific goals or intentions, and therefore decisions do not have to be derived from an individual’s previously expressed preferences (although known preferences may be considered as contributing to establishing what might be in that person’s best interests, where this might be a relevant factor). Moreover, the focus on best interests avoids the assumption made by the substituted judgment approach that preference equates to choice.

Reflecting such varied considerations, Brock (1993) outlines a hierarchical application of the two doctrines that prioritizes substituted judgment. According to this, where there is sufficient information available to support a substituted judgment, that should be the preferred approach. If, and only if, such information is lacking, a best interests approach should be adopted.

## Justifying proxy consent in research

If the conventional notion of first-hand, contemporaneous consent is seen as the ideal model, the onus on the advocate of proxy consent is to demonstrate that this is a morally justified alternative. So, if the essential function of consent it to make permissible actions that would be impermissible in the absence of such consent (Walker 2018), we must demonstrate that consent obtained from a proxy can fulfil the same function. One way of doing so would be to argue that what we have called the ‘standard’ grounds for proxy consent apply in the research context just as they do in the clinical context. Failing this, an alternative, or additional, justification must be adduced.

Before considering in normative terms what form such justification might take, it is worth outlining a few ways in which research and clinical practice differ circumstantially as regards consent. In clinical practice, whilst sometimes the options at stake are either having treatment or not having treatment, very often the decision has to do with alternative treatments—a choice between treatments rather than a choice as to whether or not treatment takes place. In research, however, the choice is generally of this latter type—should the individual enter a research study or not? A further contrast follows. In clinical practice, there may be reason, at least for the clinician, to believe that one treatment option has a more favourable harm-benefit ratio than another and, moreover, that treatment is a better option than no treatment.^8^ A consent decision of this type is centred on gaining, or at least not forfeiting, clinical benefit and therefore has, *a priori*, some form of clinical imperative. In clinical research, however, the principle of equipoise dictates that each of the treatments to be tested should be regarded, *a priori*, as therapeutically equivalent (Freedman 1987); thus, if one of the trial treatments is current standard care, there should be no prior expectation of gaining additional clinical benefit through entry to a trial. Furthermore, if some other treatment than those being tested were optimum for a particular patient, the trial treatments would not be indicated for this individual and no choice as to participation would be required. To this extent, the implications of a decision on research participation do not, or at least should not, revolve around clinical benefit, unless perhaps the optimum treatments were, for unavoidable reasons, only available within the trial.^9^

One response to the challenges posed by proxy consent in research would be simply to rule out all types of study in which participants themselves are unable to provide consent. This can be rejected straightforwardly, as it would prohibit research in areas where relevant data can only be gathered from those with the condition that renders them incompetent and, hence, where the only means of gaining a form of consent would be through a surrogate (e.g., acute head injury, dementia, neonatal medicine). A blanket ban on such trial participation would potentially be to the huge detriment of our understanding of certain widespread conditions or of developing improved care and treatment for significant parts of the population. It would also make the strong and unwarranted assumption that an inability to provide consent should be construed as if it were a refusal of consent.^10^ Additionally, such an assumption would also contribute to a justice-related concern, whereby those who would wish to be included as participants in research are excluded as a group. It is notable that the recent revisions to the Declaration of Helsinki require access to participate for underrepresented groups in research (WMA 2024, para. 13), as well as making allowance for weighing the harms of exclusion against the harms of inclusion for vulnerable groups (para. 19). There may be, for example, circumstances where persons unable to consent have made clear their wishes to participate in research, or cases where they have made clear indications as to their values and preferences in favour of certain types or areas of research, yet members of such a group are widely excluded from participating due to assumptions about consent. This is not simply to throw away the important concerns and safeguards that have long motivated the exclusionary approach to participation in research for those unable to consent. What is required, instead, is a way of establishing proxy consent as normatively acceptable in clinical research so that we can gain the safeguards arising from consent and also fulfil the social contract whereby we value consent and do not treat research participants as a ‘mere means’ to a researcher’s ends.

We will first address this issue in terms of the way in which the two ‘standard’ doctrines previously outlined—substituted judgment and best interests—are conventionally construed and the extent to which they can effectively ground proxy consent. We will then examine alternative or supplementary justifications in order to determine whether there is another approach to proxy consent suitable for the research setting. Ultimately, as we shall argue, it is the adoption of an intuitionist approach that will provide the appropriate nuanced balance of considerations for a proxy to provide consent by allowing a range of *prima facie* values to contribute to the proxy consent process, thereby facilitating participation in research while engaging in due consideration of appropriate safeguards and patient values. This has the further advantage over the other available method for determining consent to participate in research following incapacity—the advance research directive—of allowing a dynamic process that can make determinations across a whole range of different types of research and types of circumstance that incompetent persons might find themselves in and that might not have been foreseeable when the advance directive was written.^11^

### Substituted judgment

We have noted earlier that a substituted judgment justification for proxy consent is based, to a considerable degree, on epistemic grounds. Knowledge of the individual’s prior expressed wishes or preferences—or inferences as to what these wishes or preferences might be, based on other information—can support a claim that a decision made by a surrogate is likely to replicate, or at least approximate, a decision that the individual would have made in those circumstances. Conversely, “[i]f the surrogate’s knowledge of the subject’s values and preferences is approaching zero, so then is the ethical validity of the enrollment decision” (Harrison 2024:468).^12^

This evidential justification may be plausible in clinical practice. Here, the patient may have anticipated the situation in which consent would be required and a proxy decision-maker (to whom the patient’s wishes would have been communicated) authorized accordingly. This means there is first-hand evidence that can be adduced to support the proxy decision. Even if the specific situation in which consent is required had not featured explicitly in prior statements or communication of intentions and wishes on the patient’s part, the person’s preferences may be inferred from a range of other communications, or actions, relating to the illness and its medical care hitherto.^13^

The proxy is likely to face a greater epistemic burden in relation to therapeutic research. It is much less likely that a patient would have indicated, while competent, intentions regarding research participation; such a situation may never have been envisaged, and even if it had been, specific wishes may very well not have been articulated. Admittedly, there may be some instances in which this might have occurred; for example, in chronic progressive diseases for which effective treatments are not yet available, patients may be well attuned to the possibility of such treatments being developed through research and may have considered, and discussed, potential participation in such studies with relatives or friends. In other instances, however, particularly in more acute or intermittent conditions, taking part in clinical research may not have been foreseen and, therefore, never raised in discussion. Furthermore, even if research participation had been foreseen, clarity as to what this would involve is usually far harder for the individual to achieve than in the case of clinical care. In particular, as clinical research often focuses on a novel intervention, the specific focus of a study such as a clinical trial is unlikely to have been foreseen, even if the more general idea of participating in such a trial had been anticipated. Additionally, what a future clinical trial would involve is difficult to determine, as specific information on this is normally only made available at the time that consent is sought. This would leave, at best, only a general, vague indication of intention on the part of the individual, prior to losing competence, to participate in a trial.^14^

An additional issue is that the challenge arising from the potential participant’s limited understanding of the nature and implications of research participation may itself be compounded by a similar lack of understanding on the part of the proxy. What evidence there may be of the person’s intentions may be hard for proxies to contextualize and act upon without themselves having a good grasp of what taking part in clinical research entails. Prior intentions are thus much less likely to provide a clear warrant for proxy decision making in research—such intentions are unlikely to have been explicitly stated, may be less easily inferred from other relevantly similar past intentions, and may be harder to contextualize than in the case of consent in clinical practice. Moreover, Crutchfield and Scheall (2019) suggest that, *ceteris paribus*, the option for which the epistemic burden is smallest is the one that there is most incentive to choose. If the proxy chooses an option on this basis, it may differ from the option that the individual concerned—who may have faced a different set of epistemic burdens—would have chosen.

There is a deep-founded, philosophical conceptual root of this issue, concerning how we can ever accurately assess the validity of a substituted judgment claim; that is, how are we to understand a decision that claims to be the one that the individual would have made, if he or she had been able to do so? What is it to interpret such a claim as true? Such claims are known as ‘counterfactual claims’, so substituted judgments are based on a form of ‘counterfactual wishes’ modelling. All counterfactual reasoning makes appeal to the notion of alternative ‘possible worlds’, where the counterfactual situation is considered to have obtained.

There have been various attempts to harness this approach specifically to explain proxy consent and counterfactual wishes.^15^ In Barnbaum’s (1999) account, there is the ‘consent world’, in which the individual has previously stated or, at least, implied consent, and the ‘actual world’, in which the individual has not given consent, so consent is subsequently requested from a proxy decision-maker. In order that the counterfactual judgment on which substituted judgment is based can function, these two worlds must be relevantly similar. As Barnbaum (1999:171) puts it: “The agent y, in the consent world, must consent to [an intervention, or its withholding or withdrawal] in the actual world for x’s actual proxy consent to be valid.” That is, it requires the consent world, in which desires or intentions for the future are stated or can be inferred, to make reference to the circumstances of the actual world in which consent is sought, in relevant ways. Given that we can conceive of many different possible worlds that are similar to the actual world, the world in which the consent takes place should be the closest to the actual world in all respects with the sole exception that, in the former, the individual consenting is able to express wishes or intentions about the proposed course of action. Where this is the case, a *prima facie* justification of substituted judgment is provided. Such a situation is one in which either an explicit or an implicit statement has been made (in the consent world) granting, or withholding, consent to a future intervention (in the actual world), such as when a patient specifically indicates that in the event of a future loss of competence a particular clinical intervention is either permissible or impermissible. In such circumstances, a proxy decision based on substituted judgment would be justifiable—the counterfactual claim about the individual’s wishes made in the substituted judgment is held to be true just because those are the wishes expressed by the counterpart of that individual in the possible world closest to this one in all other respects. This means we can reasonably hold true the claim that the individual would have consented if he or she had been able to do so.

While this gives us a foundation to appeal to the truth (or falsity) of a proxy’s substituted judgment decisions about what an individual would have decided, were they able to, the counterfactual wishes approach itself gives rise to a number of challenges, as outlined by Nagasawa (2008) and Wrigley (2011). Moreover, the research context makes certain of these challenges even more acute. Notably, one significant problem for determining consent in many situations of research participation is that the circumstances of the actual world are much less likely to have featured in the consent world. This is a feature of how we determine the truth of counterfactual reasoning using possible worlds semantics by an appeal to consent taking place in the ‘closest possible world’. The challenge in such cases is how to interpret this closest possible world, giving rise to two significant types of problem.

First, there is the concern about how different a consent world can be from the circumstances in the actual world and still legitimately be called the closest possible world—a decision based on such reasoning may be challenging, as different possible worlds that could legitimately lay claim to being equally ‘close’ might lead to different conclusions regarding consent. However, the closest possible world where the individual for whom the proxy is deciding was able to express wishes about research participation will almost certainly deviate from circumstances in the actual world to a greater degree than in the case of the closest possible world where they expressed wishes about treatment options. This has to do with the concerns we indicated earlier about the vagueness of any likely consent due to the narrower context and greater specificity surrounding clinical research studies. This, in turn, pushes the similarities with the actual world further away, as the possible world where consent did take place would involve more differences in order to create this more specific and narrow research context in which consent would take place. This makes claims about what happens in such a world more removed from the actual world and, hence, provides a more tenuous basis as to what would have been the case.

This greater removal from circumstances in the actual world also gives rise to the second, more general, concern about the use of possible worlds as a basis for determining the truth of counterfactual claims, alongside those concerns specific to the consent scenario itself. To elaborate on the point made above, if we accept that for any actual world there are multiple alternative possible worlds (Lewis 1973), there will likely be more than one possible consent world that would have equal claim to be the ‘closest’ possible world. Each of these may be relevantly similar, in contextual and circumstantial terms, to the actual world, yet might point to differing conclusions as regards consent, such that “it is not possible to determine which world we should refer to” (Nagasawa 2008:222).^16^ The result is that identifying the consent world may be challenging or even impossible, a result about which Wrigley (2011:184) claims that even upon the most charitable model of relevant similarities between different possible consent worlds, “a counterfactual wishes model will not let us distinguish between different, but plausible, judgements.” Importantly, this problem is likely to be more acute in relation to research than in relation to clinical care, given that determining relevant similarity between the consent and actual words is harder in the former case.

### Best interests

A justification for proxy consent in research based on best interests has to identify a way in which the individual’s welfare will likely be bettered through giving (or withholding) consent. This may be harder to articulate in the research context than in clinical practice. In the latter—with some exceptions, such as organ donation—consenting to participate in a clinical procedure or receive a treatment, and to incur any risk of harm that it involves, relates directly to a benefit to the person him- or herself, whose best interests are thereby readily identified.

In research, there may be no *expected additional clinical* benefit to the individual through participation [though there might turn out to be, if one treatment is shown to be superior and, even if not, there may also be other benefits, such as the ‘inclusion benefit’ in clinical research described by Lantos (1999)]. For example, and as noted earlier, in clinical trials key ethical considerations would suggest that, *a priori*, there is no greater clinical benefit to be expected from participation than from non-participation (assuming the trial is testing against an established treatment regimen). Moreover, the clinical benefits that arise from research normally flow to future patients, rather than to those who participated in the study. For example, the findings of most surgical research will only benefit participants in the very rare event that they require the tested, or a very similar, surgical procedure a second time. Even if, in a particular situation, research findings were capable of influencing the subsequent care or treatment of individuals participating in a study, assessing how this might affect their best interests is challenging. This difficulty in identifying what is in a person’s best interests is illustrated in Berger’s (2011) example of a clinical trial of a drug with the potential to improve cognition in patients with dementia. If the drug were shown to be effective, it might be assumed that an improvement in cognitive capacity in a participant who subsequently received it would be a benefit. However, by bringing about a new and possibly stark awareness of the participant’s own impairment, it might be a disbenefit, and it may be hard to determine which of these outcomes would be the case.

It is perhaps arguments such as these that impel Berg et al. (2013) to claim that proxy consent to clinical research can only be based on substituted judgment, as the absence of clinical benefit from taking part precludes the person’s best interests from being advanced. In response to this view, it can be objected that there are situations, such as chronic illnesses, in which participants’ own clinical care may subsequently be informed by the findings of a study in which they had taken part. Nonetheless, much, if not most, clinical research does not hold out direct clinical benefit for those participating, in relation to a decision not to participate, and the standard best interests justification of proxy consent is to this extent hard to sustain.

## Related sources of justification

An analysis of the doctrines of substituted judgment and best interests, as these are standardly described in relation to clinical practice, does not yield a persuasive justification of proxy consent in the research situation. In the first case, the epistemic difficulties in establishing congruence between a person’s previously expressed, or inferred, wishes and the purpose for which consent is sought are much more challenging than in the case of clinical practice. In the second case, it is much harder in research than in clinical practice to point to a way in which participation can further the individual’s interests, at least in terms of clinical benefit. To provide a robust basis for proxy consent, these doctrines may need to be construed rather differently and/or supplemented by other moral considerations.

We will consider three approaches to proxy consent that expand, and to a degree go beyond, the doctrines of substituted judgment and best interests; these are the authentic life approach (Brudney 2009), the endorsed life approach (Phillips and Wendler 2015), and the substituted interests approach (Sulmasy and Snyder 2010). In the following section, we will then examine an approach to the justification of proxy consent in the research context that can broadly be seen as an ‘argument from ought’. Finally, we will argue in favour of a more fruitful approach than those previously offered that draws on moral intuitionism.

### The authentic life approach

Brudney (2009) describes a basis for proxy consent that relies neither on autonomy—or, as he puts it, self-determination—nor on best interests. He finds an appeal to self-determination inadequate because an individual may have never gained, or may now have lost, the capacity for autonomous decision-making and we are “no longer in a position either to respect or to disrespect that capacity” (2009:34). Recourse to the best interests approach is not, for Brudney, the automatic next step once an appeal to self-determination has failed. Instead, he turns to the notion of an authentic life. Here, he describes authenticity in terms of “the capacity to be a particular self, a distinctive individual” (2009:32). This necessarily refers not simply to the person as he or she is now, but draws in a consideration of past beliefs and values and seeks a continuity with these elements of a person’s life.^17^ Elsewhere, Brudney and Lantos (2011) contrast this persistent sense of authenticity with the idea of agency that underlies an autonomous consent decision. To this extent, agency is momentary and episodic, whereas authenticity is sustained and diachronic.

On the basis of this sense of authenticity, the proxy can seek to determine what the person would *want done*, not what he or she would hypothetically *choose* at a particular point in time, as is the normal basis of the substituted judgment approach.

### The endorsed life approach

The mainstay of the standard substituted judgment doctrine is an attempt to ground proxy consent in an individual’s previous autonomous wishes or intentions. This is a particularizing approach in that it seeks a largely one-to-one correspondence between the consent decision and specific prior expressions of wish or intention. Further, as noted earlier, it relies on a logical nexus between what a person would have wanted or preferred and what he or she would have consented to.

Phillips and Wendler (2015) describe a version of the substituted judgment doctrine that they call the endorsed life approach. Here, proxy decisions are not based on an attempt to replicate decisions that the incompetent person would have made in the situation at hand. Instead, the decision to be made is the one that most effectively reflects or promotes the life that the individual valued for him- or herself. Phillips and Wendler (2015:725) note:

Even patients who never indicated how they wanted to be treated in the event of incapacity likely offered indications of the sort of life they valued for themselves, or the kind of treatment they regarded as good or bad for them.

They further point out that respecting a person’s autonomy in this way does not involve an appeal to counterfactuals, at the level of specific decisions that this person would make, but instead takes account of the “course of life that the patient endorsed while competent, their values and dreams, to continue to determine the course of their lives” (2015:725). This is less epistemically demanding than the standard substituted judgment approach. Instead of trying to respect a previously expressed decision relating to a specific situation, or inferring such a decision from relevantly similar intentions, proxy decision making can take a more holistic view of the incompetent person’s life. In this way, if treatment participation can plausibly be seen as part of the life that the person valued, consent can, *prima facie*, be granted—or, of course, denied if participation appears discordant with such a life. Whilst this approach has, in common with Brudney’s (2009), a focus on the person over time rather than on an episodic decision, Phillips and Wendler (2015:728) distinguish the basis of their approach from Brudney’s (2009) in this way:

Brudney finds this basis in the life that the individual lived, whereas the endorsed life approach finds it in the life that the individual valued for themselves, whether or not they, in fact, lived that life.

Both this approach and the authentic life approach do not collapse into the standard best interests approach because the life that the person endorsed, or the life that is regarded as authentic, need not be the one that best serves his or her interests in respect of a specific decision. Instead, they both acknowledge a ‘whole of life’ approach to decision-making: something familiar from Dworkin’s (1993) account of precedent autonomy and his distinction between ‘experiential’ or ‘welfare-based’ interests and what he terms ‘critical’ interests. These critical interests concern the values an individual holds and are often what an individual is seeking to protect when making provision for consent to be made on his or her behalf (Wrigley 2018:323).

### The substituted interests approach

In this approach, Sulmasy and Snyder (2010) adopt the hierarchical procedure of relying in the first instance on a substituted judgment approach and then, where this fails, an approach based on best interests. However, they construe best interests differently from the standard approach. Rather than a clinician, or researcher, seeking to determine the person’s best interests, these are elicited from a surrogate who knows that person:

Rather than interpreting a text or making a substituted judgment about what the patient might have wanted in imperfectly foreseen circumstances, the surrogate is asked to apply the patient’s authentic values and real interests, including the patient’s known preferences. (Sulmasy and Snyder 2010:1946)

Given our earlier concern, when outlining approaches to proxy consent, as to how various applications of the best interests approach might collapse into substituted judgment, it is important to reflect on how a similar fate does not threaten the substituted interests approach. Importantly, to avoid such a collapse, best interests need to be construed as neither the decision the incompetent person would have counterfactually made, if able to consent for him- or herself, nor simply the incompetent person’s preferences. To avoid this, a number of key differences are found in Sulmasy and Snyder’s account. One such difference is that best interests decisions are shared with the relevant clinicians, rather than purely delegated to the proxy. Moreover, the clinician is tasked with providing a description of the individual patient’s clinical situation and prognosis. This is then combined with a consideration of the patient’s underlying values, rather than trying to determine what that person’s specific preferences would be. This provides a basis for determining what would advance the good of the patient, under the particular clinical circumstances obtaining, as a joint proxy-clinician decision.

In effect, the substituted interests approach uses a combination of the two standard approaches of substituted judgment and best interests, but it is the person’s values—rather than, or in addition to, preferences—that are substituted. Equally, best interests are not determined directly—which might be seen as paternalistic—but are inferred from an understanding of the context of the person’s “relationships, authentic values, known wishes, and real interests, given the circumstances and options” (Sulmasy and Snyder 2010:1947).^18^

### Advantages of these alternative justifications

The alternative approaches to justifying proxy consent that we have reviewed above have certain advantages over the standard doctrines of substituted judgment and best interests. First, none of these alternative approaches requires the proxy to adduce specific choices on the part of the person as the basis for a decision. They thereby avoid some of the epistemic problems that the substituted judgment approach encounters. The fact that wishes or intentions in the context of research participation may previously never have been expressed is not so problematic, as a decision is made on broader consideration of the person in terms of values and the sort of life that he or she lived or endorsed. Although determining the nature of an authentic or an endorsed life may present other evidential challenges, that these are not decision-specific allows a broader body of evidence to be considered across a person’s life. Furthermore, possible concern over paternalism within the standard best interests approach is lessened. Thus, in the substituted interests approach interests are not judged solely by the proxy but are based on the person’s own values. Any paternalism that may exist will thereby be ‘soft’ rather than ‘hard’ (Childress 1982); that is, orientated towards achieving what the individual would likely consider ‘best’, rather than achieving what the proxy thinks would be best for that individual.

## An ‘argument from ought’

A further justification for proxy consent occurs in a classic paper by McCormick (1974), who defends proxy consent in a way that might be classed as teleological. McCormick frames his argument in terms of research on children, but its relevance is broader, which is important when trying to make decisions for previously autonomous individuals. He also focuses specifically on nontherapeutic research, and his justification of proxy consent points accordingly to something other than the clinical welfare of the individual participant.^19^

Rather than appealing to an epistemic modal underlying substituted judgment (what the individual *would* desire or choose), McCormick points to a deontic modal (what the individual *should* or *ought* to desire or choose) (White 1975). The starting point for his argument is the notion that there are basic values to which we are inclined and that define our flourishing as rational and social agents—the widely accepted teleological goal of Aristotelian virtue accounts governing norms of our behaviour. To this extent, they are values that we *ought* to pursue. Furthermore, they are values that would be pursued not just for the individual but also for others. Thus, for McCormick (1974:12–13), the value that attaches to life depends in part on health:

To share in the general effort and burden of health maintenance and disease control is part of our flourishing and growth as humans. To the extent that it is good for all of us to share this burden, we all *ought* to do so. And to the extent that we *ought* to do so, it is a reasonable construction or presumption of our wishes to say that we would do so. The reasonableness of this presumption validates vicarious consent.

There is, however, an important caveat in McCormick’s proposal. He emphasizes that this sense of ‘ought’ only operates in a situation which there is no “discernible risk, undue discomfort, or inconvenience” (1974:17), and he rejects the notion that the potential benefit of nontherapeutic research could outweigh, and thus justify, any such risk, discomfort or inconvenience to participants: for McCormick this would be a “utilitarian subordination of the individual to the collectivity” (1974:15). His position is teleological, but not consequentialist, so there is no question of sacrificing the individual as a means to an end for the benefit of the many.

An objection to McCormick’s model of proxy consent is that it ascribes not only moral worth but also moral agency to children. Accordingly, May (2007:242) has argued that those who are noncompetent do not have moral obligations: “Precisely because they are noncompetent, they are *not moral agents,* but they *are* persons who ought never to be used as a means to an end extrinsic to themselves” (original emphasis). May directs this objection at both children and other noncompetent individuals. It is perhaps fair to reject children as having moral obligations, but for those who have previously been competent, the fact that they are currently noncompetent does not necessarily obviate considerations (such as receipt of medical care built upon previous research or some form of prior commitment) that could ground an obligation to participate in research (John 2009). Harris and Holm (2003) recognize such an obligation and argue that children are, at least potentially, moral agents and we can assume that they would therefore wish to do what is right. On this basis, they conclude, on similar lines to McCormick, that even in the absence of any benefit to a child, a parent can justifiably give proxy consent to that child’s participation in clinical research.

Perhaps a more fundamental objection to McCormick’s and Harris and Holm’s standpoints is that they do not so much justify proxy consent as diminish its relevance. Consent operates to protect choices that are not subject to obligation. Hence, if it is argued that an incompetent person is obligated to participate in a study and consent is then sought from a proxy, the granting or withholding of such consent would seem to be more an affirmation or a denial, respectively, of this obligation than a protection of the incompetent person’s counterfactually ascertained choice. In this connection, the National Commission for the Protection of Human Subjects of Biomedical and Behavioral Research (NCPHSBBR 1977) argue that the justification for involving children, and by extension other noncompetent persons, in research would rest upon the strength of their purported obligation to participate, and proxy consent would therefore lack a justificatory function:^20^

If a child ought to do something and the obligation justifies the child’s doing that thing, then the consent of his parents could neither validate nor invalidate his participation. Parental consent is simply irrelevant to the justification for involving the child in research. (NCPHSBBR 1977:103)

On this basis, proxy consent serves to protect the welfare of child, or other person for whom it is sought, in relation to possible risks incurred through taking part, rather than justifying the decision as to that individual’s participation.^21^

## An approach based on intuitionism

The final approach to be considered is one that moves away from attempting to justify proxy consent by focusing on a single principle or value, such as autonomy (e.g., the substituted judgment approach), welfare (e.g., the best interests approach) or obligation [e.g., the arguments of McCormick (1974) and Harris and Holm (2003)], and instead takes a slightly more complex ethical model that embraces a wider array of values and motivations involving consequentialist reasoning together with deontological duties, akin to the intuitionist approach to ethics adopted by Ross (1930, 1939).^22^ Rossian intuitionism is known for rejecting absolute deontological principles and recognizing that there are a range of motives that have moral worth, including, e.g., personal relations such as having devotion to another person, and a range of basic yet defeasible moral principles and non-instrumental goods guiding our actions and motivations. Taking its cue from Prichard (1912), his approach also does not rely on a direct appeal to general deontological principles, but allows right- or wrong-making attributes of a particular act to be determined by “direct insight” (Ross 1939:171). This has given rise to the notion of *prima facie* duties and obligations, which give us a moral reason to act unless there is some other, additional reason, of sufficient strength, to do otherwise.^23^ Importantly, there can be no *a priori* ranking of such duties and we therefore cannot tell in advance which will be the most important (Dancy 1991); we have to examine the morally relevant features of the particular situation in which we find ourselves.

Adopting such an intuitionist approach allows the proxy to consider both the duties one has to the individual and duties of broader benefit to society. This might involve the proxy considering what values and goals the individual had in life, together with a reflection upon the proxy’s commitment to make decisions based upon those. But it also allows the proxy to consider a wider form of balance that might include the benefits to society that research participation would bring and whether they might consider the incompetent individual to have any *prima facie* obligations towards research participation, weighed against the likely risks of research participation and any broad life values.

Such an approach, by considering a plurality of duties and motivations, would result in different decisions for different types of research participation. Decision-making by the proxy in this regard would mean reflecting upon the research’s value and risk assessment from both an objective (consequentialist) aspect and a subjective element that considered how those elements of value and risk aligned with a person’s previously expressed preferences, life goals and ambitions. Balancing these would mean, for example, that high-value but high-risk participation in therapeutic research would be easier to endorse if the value of the research also aligned with the individual’s broad values (and not just as a direct assessment of that person’s own, or other people’s, potential clinical outcomes). So, if the research was advantageous to a group or a cause to which the individual had strong connections or value-orientation, that would be in its favour. However, if it did not align with these values, or if the evaluation were neutral in terms of the individual’s own life goals, etc., then a much more substantial case would need to be made to move forward with a coarser ‘objective’ consequentialist analysis of the potential value versus the potential disbenefits of the research in order to determine whether the right balancing of obligations and other morally important motivations to participate was in place. This gives us Rossian intuitionism in application, where our moral duties are seen as imposing only a *prima facie* reason to accept or adhere to them. As such, they are a form of provisional consideration that can be overridden upon reflection on these intuitive moral duties and their context. This is not to say that some duties are anything other than extremely strong considerations, and countervailing reasons would have to be very substantial indeed for them to be overridden. However, they offer Ross’ common-sense approach to moral deliberation, allowing a rejection of absolute duties that cannot be deviated from under any circumstances, while adding depth to consequentialist thinking that would otherwise only recognize one basic moral good. Both the Kantian deontological approach and the utilitarian approach, according to Ross (1930, 1939), offer a far too simplistic approach to moral deliberation that fails to capture many of our moral intuitions when it comes to moral decision-making in complex circumstances.

In this way, following Ross, the stringency of the consent requirement, and thereby the legitimacy of using a proxy, can be gauged in terms of the specific situation at hand, taking account of proportionality between the importance of the research objective, the nature and procedure of the research (including its potential for risk of harm), and the availability of alternative means of participant inclusion (Hermerén 2012). We would therefore be extremely unlikely to be in a position where such intuitionist deliberation would advocate entering an incompetent person into nontherapeutic research that carried more than minimal risk without there being the potential for benefit of at least equivalent magnitude to the same individual—yet this would not be impossible. Should there be an overwhelming and clear set of values that the incapacitated individual was known to have held, that might go some way to mitigating some risks. Similarly, these decisions can include additional weighting given to benefits accrued by others, such as those with the same condition that has led to the incapacity of the individual. Even so, such risks could only be countenanced in a limited way following considerable reflection and balance, not least to ensure two very strong duties are honoured—that of avoiding unnecessary harm and that of avoiding exploiting the incompetent individual. Nevertheless, there are interesting additional benefits that arise from such intuitionist thinking, as this might also lend itself to the authentic life approach, given the onus on considering whether and how the value of the research aligned in any way with the meeting of goals, values, etc. that incompetent individuals themselves were deemed to have during their life.

### Case study

The following vignette, adapted from one described by Dunn et al. (2011), illustrates the way in which intuitionism might operate in relation to justifying proxy consent:

Arthur Riley, who has a seven-year history of Alzheimer’s disease that is now at an advanced stage, is a potential participant in a placebo-controlled trial of a drug developed specifically to target behavioural and mood symptoms in this disease. Having shown some benefits in patients with mild or moderate disease, the drug is now being tested in more advanced disease for the first time. Arthur is a widower with no children and his sister-in-law, previously appointed as proxy decision-maker in the absence of immediate family, has been approached for consent, as he is not competent to consent for himself.

In addition to physical and psychometric tests, the trial would involve blood and urine tests. Of the known potential risks of the drug, some (fatigue, headache, dizziness, and discomfort during venipuncture) are considered to be fairly probable but minor, whilst other potential risks (cardiac arrhythmias, abnormal liver function) are more serious and, whilst considered to be of very low probability, cannot be ruled out. Prior to his illness, Arthur worked as a physician and had conducted fund-raising for the British Heart Foundation in his spare time, but is not known to have expressed specific wishes or intentions regarding future research participation.

Both the substituted judgment approach and the best interests approach face a challenge in reaching a convincing conclusion on whether or not consent should be given on behalf of Arthur owing to their privileging of either autonomy or beneficence, respectively. In seeking to reach a decision, advocates of substituted judgments will discount, at least partially, beneficence-based concerns and will need to rely considerably on epistemic considerations to ground a decision based on Arthur’s counterfactual wishes. However, there is very little evidence as to what Arthur would want to happen regarding consent, as he has not previously expressed any wishes in this respect, such wishes cannot be inferred with any great confidence from what we know about his previous life, and he has no immediate family who might be best positioned to express such wishes on his behalf. Any decision will therefore be epistemically uncertain. Meanwhile, those espousing the best interests approach—who will correspondingly discount, at least partially, autonomy-based concerns—will need to point to ways in which Arthur’s welfare will be either enhanced or undermined by participating in the study, and will need to acknowledge that any clinical benefit he might gain would be symptomatic rather than disease-altering. As we have argued earlier, clinical benefit is hard to determine in relation to research participation, and particularly so in this case given that the potential benefit of the new drug is as yet uncertain in advanced disease. Accordingly, neither of these two approaches is on a sure decisional footing in this scenario. We have earlier suggested that the authentic life (Brudney 2009), endorsed life (Phillips and Wendler 2015) and substituted interests (Sulmasy and Snyder 2010) approaches have certain advantages over these orthodox approaches, but they are restricted by the same monism with respect to values.

A core advantage of the intuitionist approach in this case is that it can reach a conclusion based on a contextual consideration of the relative weight of the full range of competing *prima facie* values or obligations at play, without a prior privileging of any one such value or obligation over others. This form of value-pluralism means that intuitionism can, in this case, take full account of values derived from respect for autonomy and beneficence on an equal footing. Moreover, it can accommodate other values, such as the broader benefit to society of research into a disease with very considerable individual and societal burden, and for which there is very little possibility of gaining insight or therapeutic benefit by extrapolating research findings from other conditions. Here, intuitionism would be able to take these broader benefits into account regardless of whether the benefit is construed in consequentialist terms or in terms of some notion of social obligation. Within the substituted judgment and best interests approaches, however, a consideration of societal benefit can only exert purchase on the consent decision to the extent that it can be aligned with an inference as to what Arthur’s wishes might plausibly be or a judgment as to what constitutes his interests: both of which we have shown to be equivocal. Other approaches, such as those advanced by McCormick (1974) and Holm and Harris (2003), have this idea of societal benefit at their core, but in much the same way discount other values.

So, in the example case of Arthur Riley, a proxy adopting an intuitionist approach could reasonably make a decision to consent to Arthur’s inclusion in the trial in a way that accommodates not only the values that gave only a limited or equivocal reason for consenting Arthur to participate on either the substituted judgement or best interests approach, but also the pressing societal need to advance our understanding and treatment of Alzheimer’s disease. There is a good societal reason for Arthur’s participation in the research together with some slight indications based on a weak epistemological account as to his willingness to consent, had he been able to do so, as well as the possible but uncertain therapeutic benefits he might gain from the participation. These considerations combine to give a stronger moral motivation to make this decision to consent than would have arisen from other approaches to proxy consent that tend towards value monism to ground the decision. Some alterations of detail in this case—e.g., in relation to a different severity and/or probability of potential side-effects, an altered likelihood of direct therapeutic benefit, additional information regarding Arthur’s likely wishes, commitments or value orientation, or changes in the proxy’s understanding of the implications of enrolment—might tip the balance towards a different decision. However, with its broader embrace of relevant values, intuitionism is the approach best positioned to incorporate these additional contextual alterations.

## Conclusion

The use of a proxy to make decisions on participation in clinical research should support the fundamental normative question as to what we should decide for an individual who has lost capacity. There are certainly questions about the methodological approach that a proxy could take in order to make these decisions, yet the two most prominent approaches—substituted judgment and best interests—both fail to capture a complete picture of individuals’ wishes, their broad values, and what might benefit them, against a wider backdrop of one’s role in society. The other approaches we have considered also have shortcomings; although more nuanced than the standard justifications, the authentic life, endorsed life and substituted interests models have the similar limitation of resting largely on a single moral value, whilst McCormick’s (1974) and Harris and Holm’s (2003) approaches seem at best to dilute the meaning of consent by subordinating it to considerations of obligation.

The intuitionist approach has greater scope by virtue of eschewing monism and encompassing a range of preferences, values and other moral considerations. The various elements combining in an individual’s life are not incommensurable, as one’s wishes can reflect values that speak directly to one’s role in society and how participation is seen as a benefit to oneself. Hence, one can express wishes to altruistically benefit society: to live a life where social responsibility and the desire to ‘give something back’ play a fundamental role. For the intuitionist, it is all these factors, balanced against one another, that set out the various moral motivations for participating in a clinical study and which, once compared, should indicate what we have greater moral justification in doing in a particular situation. For proxy consent, such an approach can still build in safeguards, such as respecting clear wishes not to participate. However, even then, an intuitionist approach might consider other *overwhelming* moral motivations to tip the balance in favour of research participation. This avoids the most extreme cases of incompetent persons having endorsed in advance decisions that a common-sense analysis would suggest they would not take were they able to appraise such a situation contemporaneously. It also allows proxies to be used in their most useful sense—that of being able to make considered and dynamic judgments to establish decisions on behalf of the incompetent person. On these grounds, a decision to grant or withhold participation in a clinical trial for an incompetent individual could be made not on a speculative basis but on the basis of having the strongest overall moral motivation to support that decision on behalf of another.

## Footnotes

1 In some instances, a person who is not competent to give or refuse consent (e.g., some children) may nonetheless be able to provide assent or dissent, and thereby make some contemporaneous contribution to the consent decision. This is an important supplementary means of safeguarding the welfare of such vulnerable groups and determining their preferences, such as a desire to participate in the research, as well as a means of indicating any discomfort or harms they might experience as a result of participating. It has become a standard expectation of research practice that assent is sought, and dissent respected, in such cases, such as specified by the Declaration of Helsinki (WMA 2024, para. 29). However, we exclude such cases from our discussion, as we are focusing specifically on the role of the proxy in the consenting process for cases of individuals wholly incompetent. It remains an interesting further issue how to utilize proxy consent in cases where the individual is able to either assent or dissent; Karlawish (2007:615) discusses different interpretations of the notions of assent and dissent, which he regards as hitherto “undifferentiated concepts.”

2 The notion of intention is important here. This distinction is not based on the contingent matter of whether a participant did nor did not subsequently benefit from participating, but on whether or not the study was originally designed with therapeutic benefit in mind.

3 See Hughes et al. (2010) for a discussion of these background issues that have given rise to much discussion as to the need for consent and the possible exceptions to it in research.

4 Harrison (2024) suggests that what is granted by the proxy is better thought of as ‘permission’, as it does not meet the definitional criteria of either hypothetical or actual consent.

5 Post (2003), writing in relation to Alzheimer’s disease, takes a rather different stance on this question, and proposes an alternative, less restrictive criterion for permissible risk of harm: that of ‘maximal potential risk’. He argues that, on the condition that the level of maximal potential risk is clearly defined and subject to thorough monitoring, proxy consent may in principle be justifiable in nontherapeutic research that carries more than a minimal risk of harm. See Karlawish (2007) for a discussion of standards of research risk in research on cognitively impaired adults, and see Weijer (2000, 2001) and Weijer and Miller (2004) for an analysis of risk in relation to therapeutic and nontherapeutic ‘components’ of clinical research.

6 Individuals may indicate their wishes for the future through an advance directive. A formal advance directive is not strictly a substituted judgment, as the individual’s original consent is directly invoked rather than being substituted with some other form of consent (Wrigley 2011). Accordingly, advance directives and similar explicit prior statements of choice are placed by some under a separate heading from substituted judgment (Veatch 2003; Brock 2007; Mazur 2012). An important difference between an advance directive and a substituted judgment more broadly is that the former, by definition, takes account of future noncompetence whereas the latter may rest upon previously expressed desires or preferences that were not framed in terms of a loss of competence. The fact that these preferences will be a less reliable guide when not framed in this way may be why Buchanan and Brock (1989:84) claim that a substituted judgment should be based on what “the patient would choose if the patient were competent and aware both of the medical options and of the facts about his or her condition, including the fact that he or she is incompetent.” We do not focus on cases of advance directive or other forms of prospective authorization as the consent decision has, in effect, been made previously, and leaves little role for a proxy at this time.

7 Similarly, Buchanan and Brock (1989:116) argue that a preference is a “pro-attitude” and that the “contemporaneous choice of a competent individual under conditions of informed consent is an act of will, not the mere expression of a preference.”

8 A favourable harm-benefit ratio would obtain where the clinical benefit likely to be produced by an intervention outweighs any possible countervailing harm that might occur through side-effects or other adverse consequences of that intervention.

9 A decision to participate may involve different *types* of clinical benefit. Two novel treatments being tested ‘head-to-head’ in a randomized trial might each be thought to have greater potential to alleviate symptoms or effect a cure than the standard therapy available outside the trial. However, the trial interventions may also potentially have more severe side-effects than standard therapy. Nonetheless, whilst a particular patient might have a *preference* regarding the type of benefit that might be gained either within or outside the trial, there should be no *objective* sense in which either participation or non-participation is clinically preferable.

10 Johansson and Broström (2016) argue on several grounds that a ‘false positive’ (wrongly assuming the giving of consent) is more serious an error than a ‘false negative’ (wrongly assuming the refusal of consent), but with specific regard to the counterfactual wishes of the participant it is not clear that one error is more worrisome than the other.

11 The challenges facing the use of advance research directives have been addressed by Scholten et al (2018), and their discussion only very briefly touches on the use of proxies in this context. Although it is not the focus of this paper to offer a direct response to Scholten et al., our position is that the approach to proxy consent we outline here is consistent with the use of advance research directives and would resolve many of the challenges that their use faces.

12 This suggests that a surrogate who is relationally close to the individual is, in principle, a stronger source of such information than one who is relationally more distant. However, Hardwig (1993:2) cautions that surrogates who are relationally close are thereby more likely to have interests of their own at stake: “‘Knowledgeable’ about patient wishes usually means ‘close,’ but close almost always means having interests of one’s own in the case. ‘Disinterested’ usually means ‘distant,’ and distance usually brings with it less real concern, as well as lack of the intimate knowledge required to render a reliable substituted judgment.” Kitts and Smolenski (2025:112) further argue that one cannot assume that a surrogate appointed on a basis other than nomination by the individual is necessarily one that the individual would wish to act in this capacity, such as by being “a family member with whom they are not close, a spouse from whom they are separated, or siblings with whom a relationship is tense.”

13 Gutheil and Appelbaum (1983) are, however, sceptical of the evidential basis of substituted judgment even in clinical care and for this reason favour a best interests approach.

14 It has been argued by Scholten et al (2018) that the use of advance research directives (ARDs) on a *type* standard (i.e., indicating a broad kind or area of research in which the subjects of such ARDs are willing to participate), rather than a *token* standard (i.e. specified clinical trials), would allow incompetent individuals the opportunity to provide a clear indication of their wishes and an additional safeguard for participation in a clinical trial. We certainly agree with this. However, it is not established that such an additional safeguard is an ethical requirement where a properly appointed proxy has also been appointed. Moreover, even with such an ARD in place, Scholten et al. acknowledge that a proxy would still be required for consent to participate to be established, as the proxy would still need to determine whether the trial being considered falls under the type of trial indicated in the ARD and, additionally, whether it is compatible with the incompetent person’s values. Such ARDs might therefore help with the epistemic burden on the proxy but would not eliminate it, as the nuanced and flexible aspects of decision-making can only be derived from the proxy and not the ARD.

15 A series of papers, each addressing the problems raised by the previous account of counterfactual modelling, form the heart of this debate. See: Wierenga (1983); Barnbaum (1999); Nagasawa, (2008); and Wrigley (2011).

16 Discussing the similarity of worlds, Lewis (1973: 91) notes that this “consists of innumerable similarities and differences in innumerable respects of comparison, balanced against each other according to the relative importances we attach to those respects of comparison. Insofar as these relative importances differ from one person to another, or differ from one occasion to another, or are indeterminate even for a single person on a single occasion, so far is comparative similarity indeterminate.”

17 Similarly, for Miller (1985:243) authenticity “means that an action is consistent with the attitudes, values, dispositions, and life plans of the person. The rough idea is that of acting in character.”

18 It is also worth noting that this approach is very similar to that of the endorsed life approach, as Sulmasy and Sulmasy (2015) subsequently point out, though whereas Phillips and Wendler (2015) present the endorsed life as a variant of the substituted judgment approach, Sulmasy and Snyder distance their method from this description and present it in terms of interests.

19 In a subsequent paper, McCormick applies his arguments to the specific case of fetal experimentation (McCormick 1975).

20 The Commission reject McCormick’s substantive argument: “The form of McCormick’s argument is: if one ought to do it, then consent may be validly assumed. But consent is precisely what may not be assumed even if one ought to do it” (NCPHSBBR 1977:103). They thereby accuse McCormick of begging the essential question.

21 Similar to May (1974, 1976, 2007), Ramsey (1976, 2002) is critical of McCormick’s position, taking a strongly prohibitive stance on the involvement of children in nontherapeutic research. McCormick (1976) defends his position against some of Ramsey’s points of criticism. Mazur (2012) summarizes and analyses the standpoints of these three writers, and notes that May’s initial position later became slightly more permissive.

22 Ross’s substantive moral theory could not be described as consequentialist – it was in part a reaction against utilitarian consequentialism, which for him “over-simplifies the moral life” (Ross 1939:189) – but the underlying notion of accommodating a plurality of potentially competing moral values lends itself to considering consequentialist values alongside more deontological ones.

23 Of course, we are not the first to make use of aspects of Rossian intuitionism, with Beauchamp and Childress’ (2009) four principles approach popularizing an ethical framework approach that utilizes *prima facie* moral principles.
